# Additive Manufacturing of Alloys and Composites

**DOI:** 10.3390/ma16030992

**Published:** 2023-01-21

**Authors:** Haokai Dong, Haizhou Lu, Chao Zhao, Lehua Liu

**Affiliations:** 1 National Engineering Research Center of Near-Net-Shape Forming for Metallic Materials, South China University of Technology, Guangzhou 510640, China; 2 School of Mechanical and Automotive Engineering, South China University of Technology, Guangzhou 510641, China; 3 School of Mechatronic Engineering, Guangdong Polytechnic Normal University, Guangzhou 510665, China; 4 School of Materials Science and Engineering, Huazhong University of Science and Technology, Wuhan 430074, China

The emergence and development of high-performance materials have benefited from the revolution in modern manufacturing technology, in which additive manufacturing (AM) is the most representative over the last four decades. AM, also known as 3D printing, refers to a family of layer-upon-layer building technologies capable of producing geometrically complex engineering parts with a short lead time [1]. Compared to the conventional processing route, AM can provide more design freedom and flexible manufacturability, which has played an increasingly vital role in many custom fields such as patient-specific implants for medical application and the complex hollow structure of jet engine parts.

Nowadays, AM is widely used to fabricate metallic materials such as steels, nonferrous alloys, and high entropy alloys [2,3,4,5,6]. The repetitive high thermal gradient during AM leads to microstructural development which significantly deviates from the equilibrium condition, thereby resulting in copious metastable microstructures including hierarchically heterogeneous microstructures, multiphase constituent, nanosized precipitates and dislocation network, enabling extraordinary mechanical behaviors compared to the counterparts made by conventional methods. AM of metal matrix composites (originally invented to combine the unique properties of metals) is another hot research field which has grown in recent years [7]. AM, particularly powder-based AM methods, is proven to be a useful and versatile composite manufacturing technique that facilitates the processing of reactive primary powders for creating new materials with different constituent phases. Several intractable issues, such as weak interface bonding, cracks at the interfaces, inhomogeneous dispersions of reinforcement and residual stress induced by thermal mismatches between composing phases, appearing in conventional synthesis, could also be effectively mitigated and solved by AM.

In aiming to further develop additive manufactured alloys and composites, a full understanding of processing–microstructure–property relationships during AM is still the current scope for AM researchers, although great progress has already been made in respect of the large number of related papers published in recent years. The remaining critical challenges, including high production cost, the formation of various defects, and many inapplicable established theories in textbooks to explain physical metallurgy during AM, will continue to stimulate AM research [6], and we believe those challenges will be overcome in the near future.

In light of these contributions, the current Special Issue entitled “Additive Manufacturing of Alloys and Composites” welcomes these original research articles, state-of-the-art reviews, and perspectives on recent developments in additive-manufactured alloys and composites, which aims to provide analysis, solutions and support for creating more suitable materials for AM.
